# Quality, reliability, and engagement of COPD-related videos on Chinese short-form video platforms: a cross-sectional content analysis

**DOI:** 10.1038/s41598-026-57615-x

**Published:** 2026-06-18

**Authors:** Jiajun Liu, Juanjuan Liu, Tao Fan, Yu Zhang, Yan Lv, Jing Yang, Kehao Zhao, Ying Zeng

**Affiliations:** 1 School of Nursing, The Second Affiliated Hospital, Hengyang Medical School, Hunan Province Key Laboratory of Tumor Cellular & Molecular Pathology, University of South China, Hengyang, 421001, China; 2 School of Rehabilitation Medicine and Health, Hunan University of Medicine, Huaihua, China; 3 Division of Internal Medicine, Institute of Integrated Traditional Chinese and Western Medicine, West China Hospital, Sichuan University, Chengdu, China; 4 Department of Pulmonary and Critical Care Medicine, Chengdu First People’s Hospital, Chengdu, China

**Keywords:** Chronic obstructive pulmonary disease, TikTok, Short-form video platforms, Social media, Health information quality, Digital health literacy, Computational biology and bioinformatics, Health care, Medical research

## Abstract

Short-form video platforms are increasingly used to obtain information about chronic obstructive pulmonary disease (COPD), but the quality and reliability of COPD-related content across Chinese platforms remain unclear. We evaluated 228 COPD-related videos from Douyin, Kwai, and Bilibili using the Journal of the American Medical Association (JAMA) benchmark criteria, Global Quality Scale, modified DISCERN (mDISCERN), and Patient Education Materials Assessment Tool. Video characteristics, creator identity, verification status, content theme, presentation format, and visible engagement metrics were also analyzed. Video quality differed significantly across platforms. Douyin showed the highest transparency, reliability, and overall quality scores. Kwai showed the highest visible engagement but had lower overall quality and actionability scores. Bilibili had the longest videos and the highest understandability scores. Organization verified accounts generally achieved higher quality scores than individual verified or unverified accounts, although this finding should be interpreted cautiously because of the small number of such accounts. Visible engagement metrics, including likes, comments, saves, and shares, were not significantly correlated with medical quality scores. Together, these findings suggest that visible popularity is a poor proxy for medical quality. COPD-related digital health communication may benefit from more accessible evidence-based content, clearer source identification, and closer oversight of high-risk therapeutic claims.

**Supplementary Information:**

The online version contains supplementary material available at https://doi.org/10.1038/s41598-026-57615-x.

## Introduction

Chronic obstructive pulmonary disease (COPD) is frequently termed the “silent killer” because of its insidious progression and substantial mortality burden^1,2^. It remains a major global public health challenge and imposes a substantial disease burden among adults aged 30 to 79 years^3^. In China, the burden is particularly pronounced, with an adult prevalence of 8.6% and nearly 100 million affected individuals^4^. Macroeconomic models further predict that China will bear one of the world’s largest COPD-related economic burdens^5^. Effective COPD prevention and management depend heavily on timely recognition, risk reduction, and long-term self-management. Smoking cessation and avoidance of harmful environmental exposures are central to risk reduction^1,6,7^, whereas high-quality self-management and lifestyle modification after diagnosis are important for delaying disease progression and preventing acute exacerbations^8^. Many patients with COPD have limited disease-specific health literacy, and related studies have reported gaps in symptom perception and disease knowledge^9–11^. Such limitations may impair self-management and treatment adherence and may also weaken patients’ ability to assess the reliability of health information in digital environments^9,12^.

With the proliferation of mobile internet technology, social media has reshaped how the public accesses medical information^13^. According to the 56th Statistical Report on Internet Development in China, China had more than 1.123 billion internet users by June 2025, with a penetration rate of 79.7%^14^. Short-form videos have become prominent carriers of online health education^15^. Their audiovisual format may lower barriers to acquiring medical knowledge and complement traditional health education, particularly for populations with low health literacy^16,17^. In China, Douyin, Kwai, and Bilibili are among the mainstream short-form video platforms examined in online health communication research^18–20^. Prior studies have reported platform-level differences in health-video quality and source characteristics on Douyin and Bilibili^21,22^, while Kwai has been described as having a distinct user community profile, including strong connections with lower-tier cities and rural users^23^. Taken together, these platform-level differences suggest that COPD-related health information may vary across platforms.

Despite their accessibility, short-form video platforms also create challenges for health communication. Platform content is largely user-generated, varies widely in source credibility, and often lacks systematic professional review^19^. Platform recommendation environments may further shape how users encounter health information^24^. Creators without medical qualifications may disseminate outdated or unsupported advice, while commercial interests may promote biased or misleading information^25,26^. For COPD, which requires long-term standardized management, inaccurate information may contribute to anxiety, inappropriate self-management, or delays in evidence-based care^27^.

Previous studies have evaluated short-form videos on diabetes^28^, Alzheimer’s disease^29^, and asthma^30^, and have reported concerns regarding quality, reliability, or clinical accuracy. However, comprehensive evaluation of COPD-related short-form videos remains limited. Current COPD-related video studies remain largely limited to single platforms or relatively narrow evaluative dimensions^27,31^. Consequently, the Chinese multi-platform digital environment and cross-platform differences in COPD-related information quality remain insufficiently characterized.

Given the heterogeneity of COPD patients in age, health literacy, and urban–rural background, a systematic multi-platform evaluation of COPD-related video quality is needed. This study used standardized assessment tools to evaluate the quality, reliability, understandability, actionability, and visible engagement characteristics of COPD-related videos on Douyin, Kwai, and Bilibili. The findings may help patients identify more reliable health information, support healthcare professionals in improving digital communication strategies, and inform evidence-informed approaches to the governance of COPD-related health information.

## Materials and methods

### Study design

This study employed a cross-sectional content analysis approach and adhered to the STROBE statement (Supplementary Table S1). The objective was to systematically evaluate the quality of health information related to COPD on mainstream Chinese short-form video platforms.

### Search strategy and data collection

Searches were initiated on December 26, 2025, in Sichuan, China, using a Windows 11 computer and Microsoft Edge (version 140.0.3485.81) in InPrivate mode from the same IP address to reduce the potential impact of personalization. Subsequently, a new mobile phone number was used to register accounts on each platform.

The exact search query entered on each platform was the Chinese term “慢性阻塞性肺疾病” (chronic obstructive pulmonary disease), which corresponds to the standard Chinese medical term for COPD in clinical and disease-coding contexts. This term was selected as the sole search keyword to evaluate the search results returned by each platform in response to standardized medical terminology. Based on evidence from previous studies, content beyond the top 100 videos on these platforms has a negligible impact on analysis results because these rankings sufficiently reflect the breadth of relevant content in the field^18,20,28,32^. Following this user behavior pattern, we selected the top 100 videos returned by the default search ranking on each platform, resulting in an initial pool of 300 videos.

### Inclusion criteria

(1) The video content primarily pertains to COPD disease definition, symptom recognition, diagnostic procedures, pharmacological or non-pharmacological treatments, rehabilitation, or nursing care; (2) The video language is Chinese (Mandarin or provided with accurate Chinese subtitles).

### Exclusion criteria

(1) Duplicate content within the platform; (2) Videos requiring payment for viewing; (3) Pure English videos without Chinese subtitles or videos without audio; (4) Content entirely unrelated to COPD, such as other lung diseases or non-medical content; (5) Pure commercial advertisements, defined as videos without substantive health education content that only perform product promotion or hospital solicitation; (6) Samples with unstable data, specifically videos published for less than seven days. The exclusion of such videos aimed to avoid bias in the analysis of dissemination characteristics caused by interaction data (likes, comments, etc.) because early engagement metrics may be unstable shortly after publication.

### Data extraction

**Fig. 1:**
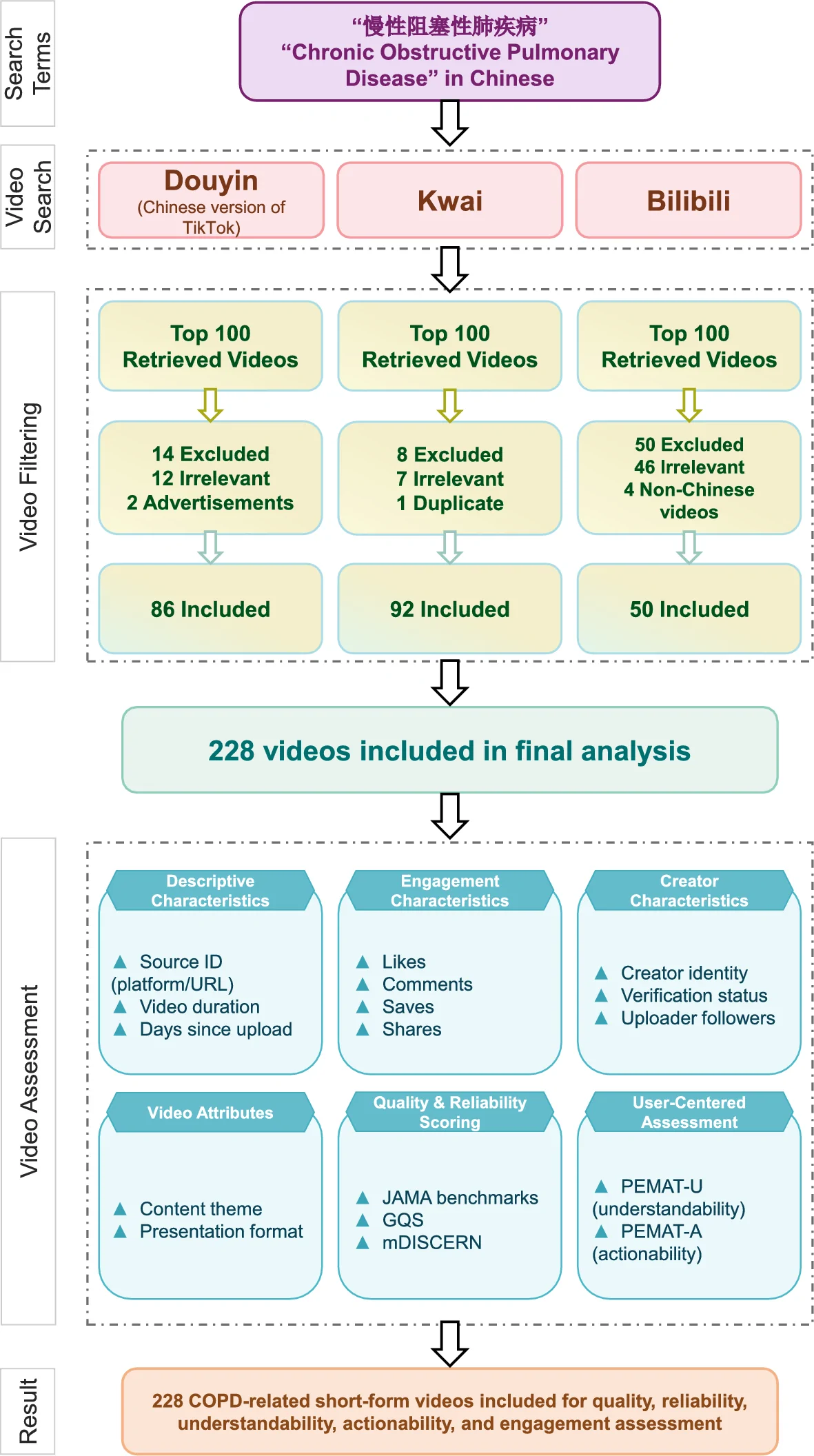
Search strategy and screening flow for chronic obstructive pulmonary disease-related videos.

The search retrieved the top 100 results returned by each platform, yielding 300 videos in total. The initially retrieved videos were screened according to the predefined inclusion and exclusion criteria. After screening, 72 videos were excluded, including 65 irrelevant videos, 4 non-Chinese videos, 2 advertisements, and 1 duplicate video. The final sample comprised 228 COPD-related videos, including 86 from Douyin, 92 from Kwai, and 50 from Bilibili. The detailed search, screening, exclusion, and final inclusion process is presented in Fig. 1. To improve transparency and reproducibility, data extraction was conducted independently by two trained reviewers (Jiajun Liu and Juanjuan Liu) using a pre-designed standardized data extraction spreadsheet. Discrepancies in extracted information or categorical coding were resolved through discussion and consensus meetings. When disagreement persisted, a third senior clinical expert (Tao Fan) was consulted for final adjudication. All video-level information and engagement metrics were extracted within a consecutive 48-h window from December 26 to 28, 2025, to reduce temporal variability in platform-displayed engagement indicators. All data were recorded in xlsx format (Microsoft Corporation).

For each included video, video metadata and public engagement metrics were extracted, including the original video URL retained internally for verification and traceability, video duration in seconds, days since upload, and uploader follower count. Standard engagement indicators, including likes, comments, shares, and saves, were recorded as platform-displayed public metrics and harmonized under unified variable names for cross-platform comparison. The platform-displayed bookmarking or saving function was operationalized as “saves” in the analysis dataset.

Key categorical variables were classified and coded according to predefined operational definitions. Creator characteristics included creator identity, verification status, and uploader follower count. Creator identity was categorized as medical professionals, healthcare organizations, or independent users, including non-clinical accounts. When professional status could not be determined, the account was coded as an independent user. Content characteristics included content theme and presentation format. Content theme was categorized as disease knowledge, clinical scenarios, or intervention strategies. Presentation format was categorized as oral monologue, visual illustration, or situational enactment. Verification status was categorized based on platform-specific certification systems as individual verified accounts (Yellow V), organization verified accounts (Blue V), or unverified accounts. Across the evaluated platforms, Yellow V generally refers to platform-verified individual accounts, such as platform-certified professionals or influential individual users, whereas Blue V generally refers to platform-verified institutional accounts, such as medical institutions, media organizations, government agencies, or other organizations. For videos containing multiple themes or presentation formats, coding was based on the dominant theme or format representing the primary educational purpose. Fine-grained communication subtypes were consolidated according to their dominant communication mode and shared visual or narrative characteristics. Detailed coding rules, operational definitions for each category, and the standardized data extraction form are provided in the Supplementary Table S6.

### Video content and quality assessment

The included videos were evaluated using four commonly used assessment instruments: the JAMA benchmark criteria, the Global Quality Scale (GQS), the modified DISCERN (mDISCERN) instrument, and the Patient Education Materials Assessment Tool for Audiovisual Materials (PEMAT-A/V)^33–36^. To facilitate interpretation, the scoring ranges and score directions were defined before analysis. The JAMA benchmark criteria assessed transparency and source traceability across four items: authorship, attribution, disclosure, and currency^37^. Each fulfilled item received 1 point, yielding a total score of 0–4, with higher scores indicating greater transparency and traceability. The GQS assessed overall educational quality, flow, and usefulness on a 5-point scale, ranging from 1 (poor quality) to 5 (excellent quality); scores of 1–2, 3, and 4–5 were interpreted descriptively as low, moderate, and high overall quality, respectively^38,39^. The mDISCERN instrument assessed information reliability using five binary items, with total scores ranging from 0 to 5 and higher scores indicating greater reliability^40,41^. PEMAT-A/V assessed understandability and actionability separately^42,43^. Items were scored as agree = 1, disagree = 0, or not applicable where appropriate, and domain scores were calculated as percentages ranging from 0 to 100%, with higher percentages indicating greater understandability or actionability. Because scoring thresholds are not uniformly defined across these instruments, the original JAMA, GQS, mDISCERN, PEMAT-understandability, and PEMAT-actionability scores were used in the primary analyses. No overall dichotomous classification of videos as high or low quality was applied. Detailed item-level scoring rubrics are provided in the Supplementary Tables S2–S5.

### Inter-rater reliability

After data extraction and categorical coding, quality assessment was conducted independently by two trained reviewers with clinical experience in respiratory medicine (Yu Zhang and Yan Lv). All included videos were evaluated using the JAMA benchmark criteria, GQS, mDISCERN, and PEMAT-A/V. Before formal assessment, both reviewers received standardized training on the scoring criteria and operational definitions of each instrument. This training included review of the detailed scoring criteria provided in Supplementary Tables S2–S5 and the standardized data extraction form, categorical coding rules, and quality assessment scoring record provided in Supplementary Table S6. Before formal scoring, the reviewers completed a calibration exercise using 10 pilot videos to ensure consistent interpretation of the rating criteria.

After this calibration, the two reviewers independently scored all included videos using the same standardized scoring form. The initial independent scores were used to calculate inter-rater reliability. Discrepancies between reviewers were first discussed in consensus meetings. If disagreement persisted after discussion, a third senior clinical expert in pulmonary medicine (TF) reviewed the relevant video content and adjudicated the final consensus score. Final consensus scores were used for descriptive and inferential analyses.

Inter-rater reliability was quantified using the intraclass correlation coefficient (ICC), based on a two-way random-effects model with an absolute agreement definition. According to the criteria recommended by Koo and Li, ICC values were interpreted as poor (< 0.50), moderate (0.50–0.75), good (0.75–0.90), or excellent (> 0.90)^44^. To assess test–retest reliability, Yu Zhang repeated the assessment after a two-week interval, and ICC values were calculated between the first and repeated ratings.

The inter-rater consistency analysis showed good reliability for all indicators, including JAMA (ICC = 0.839, 95% CI [0.792, 0.876]), GQS (ICC = 0.772, 95% CI [0.694, 0.829]), and mDISCERN (ICC = 0.804, 95% CI [0.744, 0.849]). PEMAT-understandability (ICC = 0.774, 95% CI [0.675, 0.839]) and PEMAT-actionability (ICC = 0.761, 95% CI [0.682, 0.819]) also demonstrated good consistency. Regarding test–retest reliability, the ICC values for JAMA (0.928), GQS (0.860), mDISCERN (0.906), PEMAT-understandability (0.864), and PEMAT-actionability (0.820) were all above 0.80.

### Statistical analysis

**Fig. 2:**
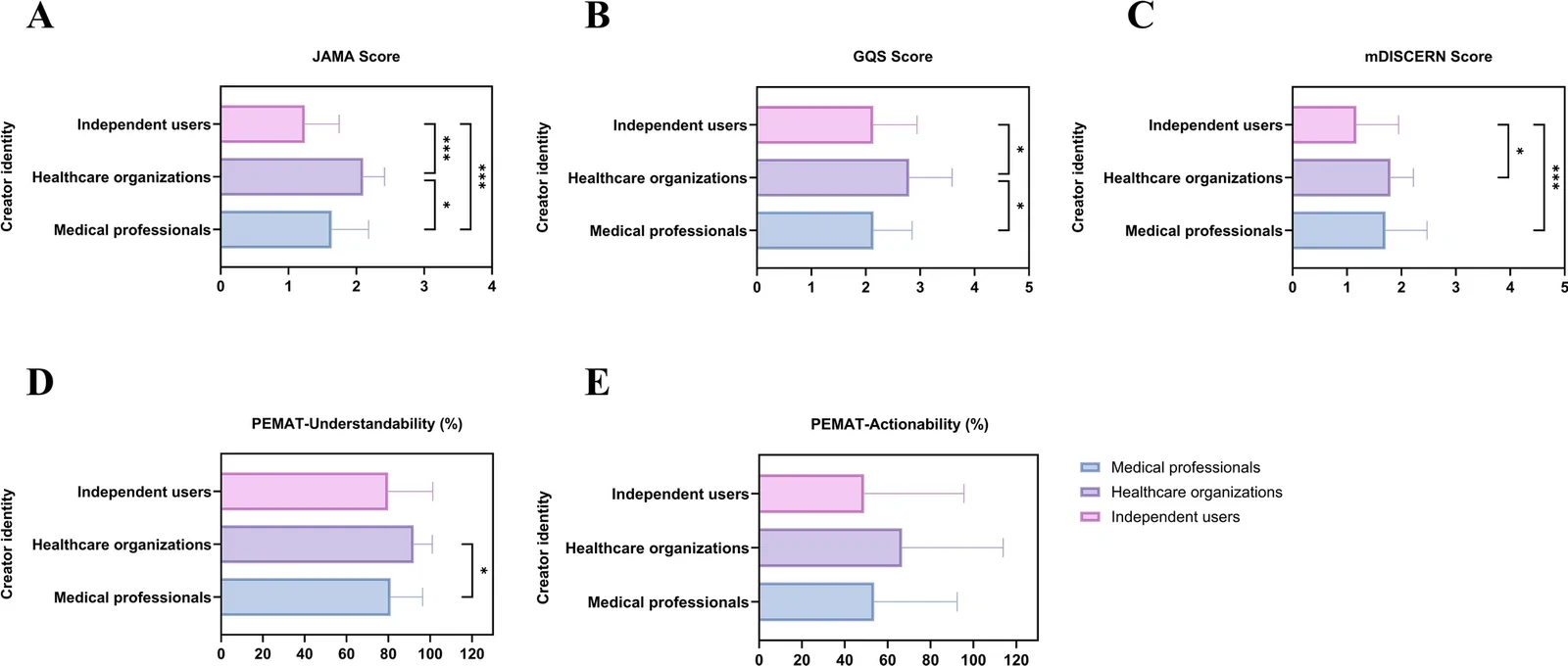
Assessment scores of COPD-related videos from diverse creator identities—including (**A**) the JAMA score, (**B**) the GQS score, (**C**) the mDISCERN score, (**D**) PEMAT-understandability, and (E) PEMAT-actionability. Pairwise comparisons were adjusted using the Dunn–Bonferroni method. * *P* < 0.05, ** *P* < 0.01, *** *P* < 0.001. JAMA, Journal of the American Medical Association; GQS, Global Quality Scale; mDISCERN, modified DISCERN; PEMAT, Patient Education Materials Assessment Tool.

**Fig. 3:**
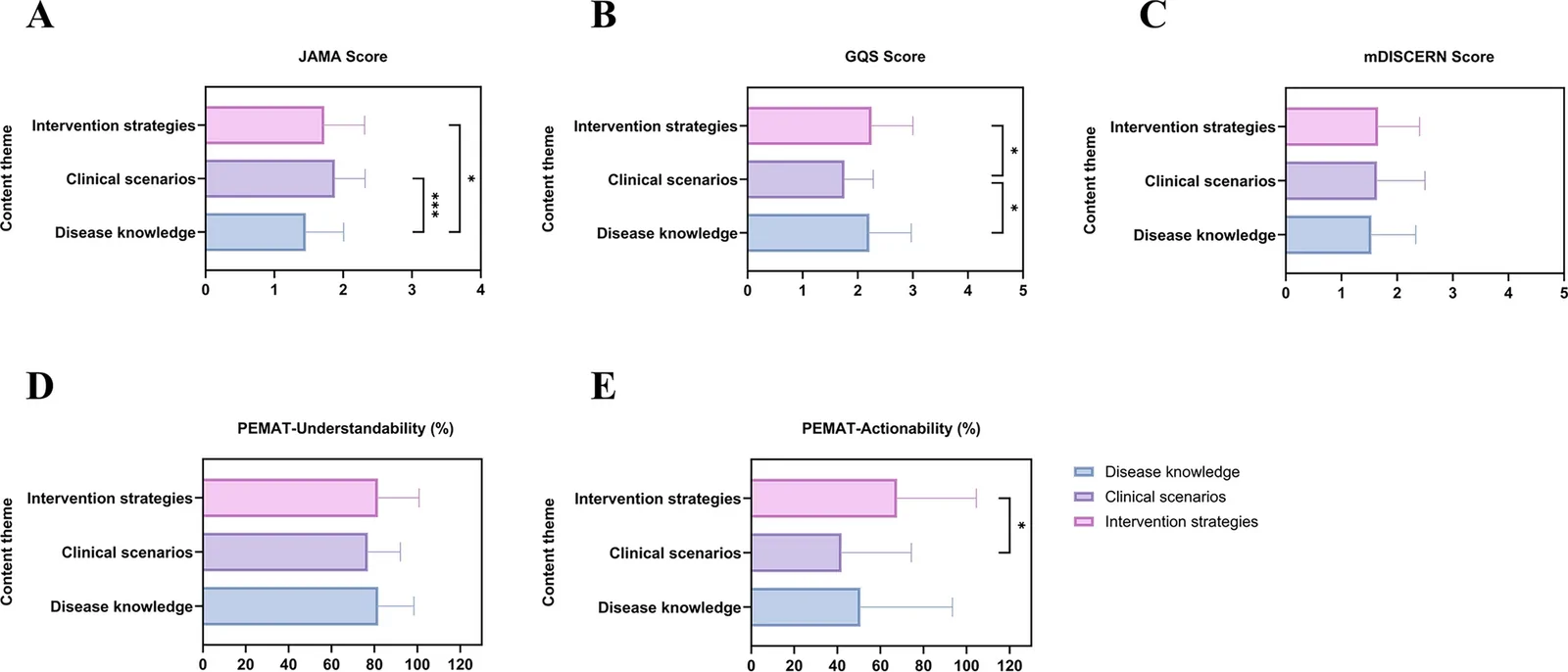
Assessment scores of COPD-related videos focusing on distinct content themes—including (**A**) the JAMA score, (**B**) the GQS score, (**C**) the mDISCERN score, (**D**) PEMAT-understandability, and (E) PEMAT-actionability. Pairwise comparisons were adjusted using the Dunn–Bonferroni method. * *P* < 0.05, ** *P* < 0.01, *** *P* < 0.001. JAMA, Journal of the American Medical Association; GQS, Global Quality Scale; mDISCERN, modified DISCERN; PEMAT, Patient Education Materials Assessment Tool.

**Fig. 4:**
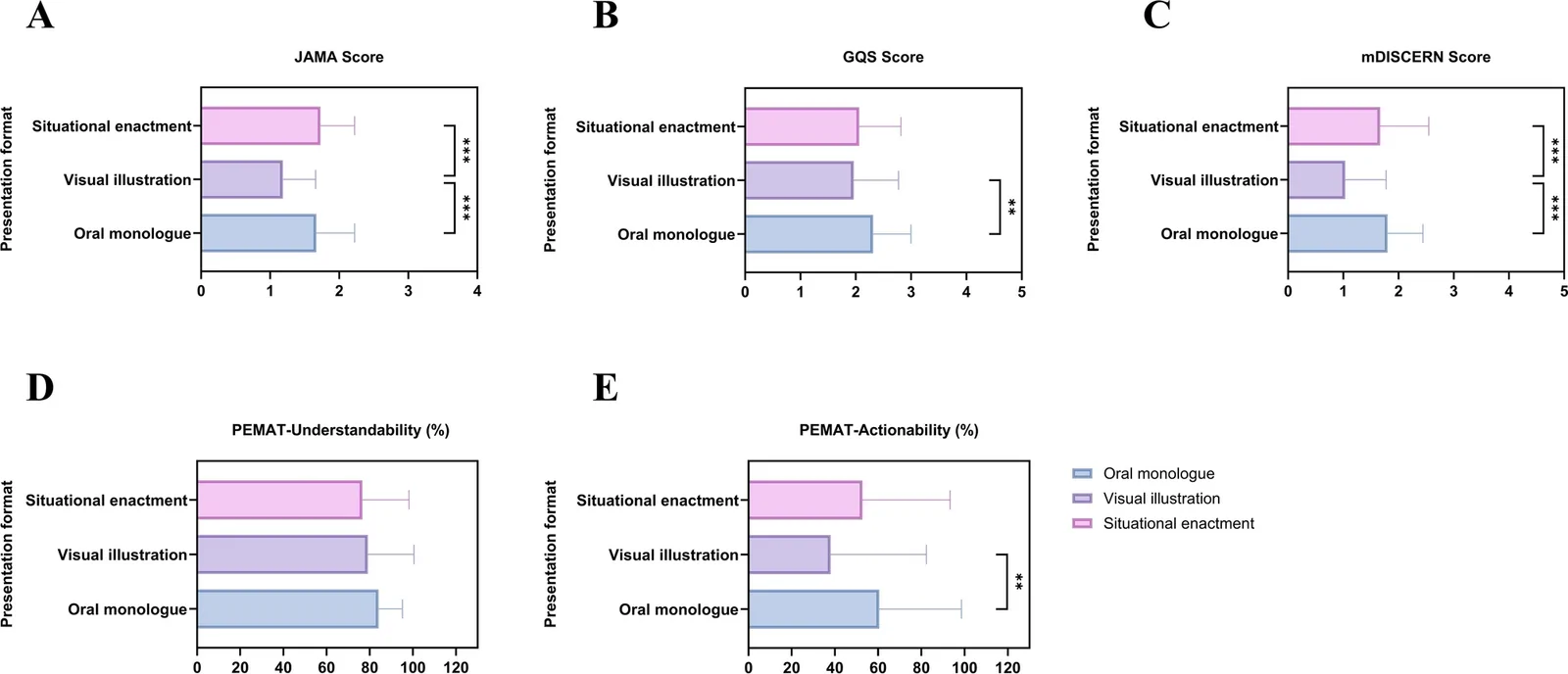
Assessment scores of COPD-related videos using different presentation formats—including (**A**) the JAMA score, (**B**) the GQS score, (**C**) the mDISCERN score, (**D**) PEMAT-understandability, and (**E**) PEMAT-actionability. Pairwise comparisons were adjusted using the Dunn–Bonferroni method. * *P* < 0.05, ** *P* < 0.01, *** *P* < 0.001. JAMA, Journal of the American Medical Association; GQS, Global Quality Scale; mDISCERN, modified DISCERN; PEMAT, Patient Education Materials Assessment Tool.

**Fig. 5:**
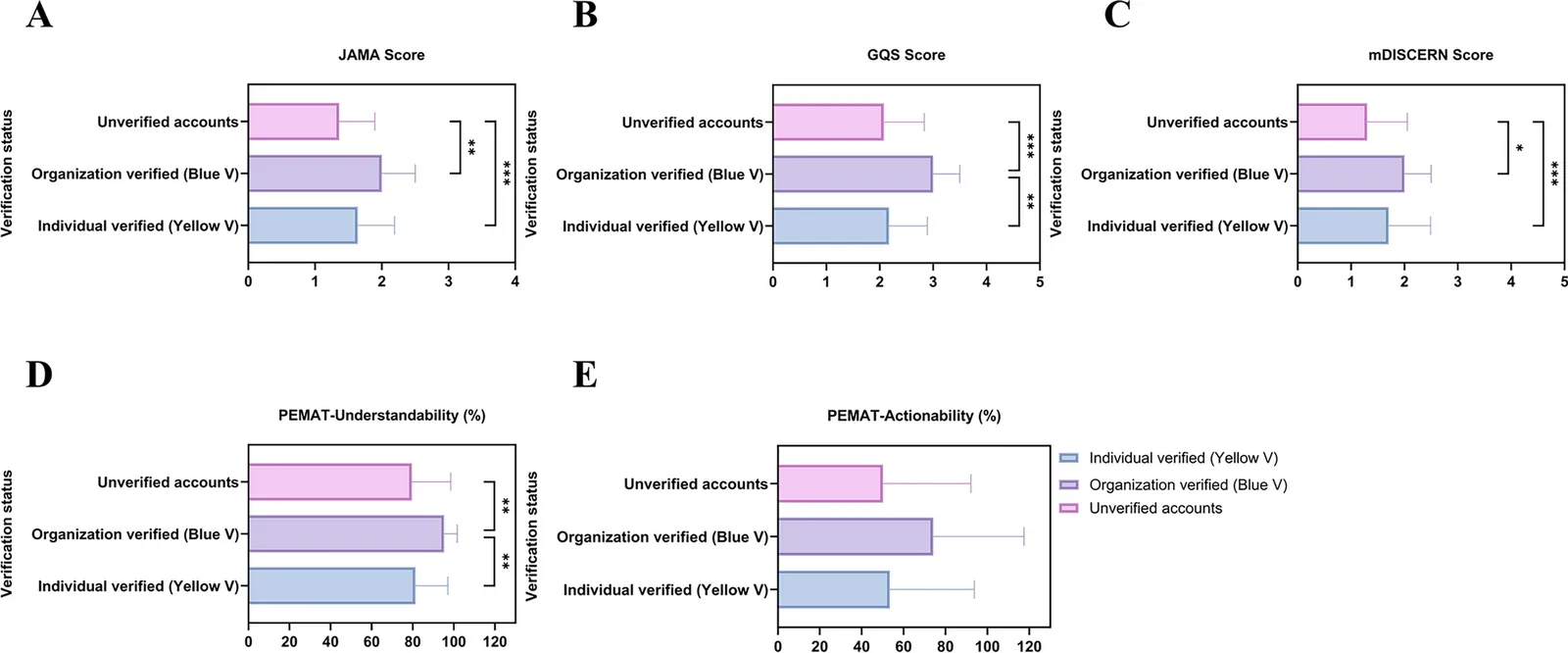
Assessment scores of COPD-related videos with different account verification statuses—including (**A**) the JAMA score, (**B**) the GQS score, (**C**) the mDISCERN score, (**D**) PEMAT-understandability, and (**E**) PEMAT-actionability. Pairwise comparisons were adjusted using the Dunn–Bonferroni method. * *P* < 0.05, ** *P* < 0.01, *** *P* < 0.001. JAMA, Journal of the American Medical Association; GQS, Global Quality Scale; mDISCERN, modified DISCERN; PEMAT, Patient Education Materials Assessment Tool.

**Fig. 6:**
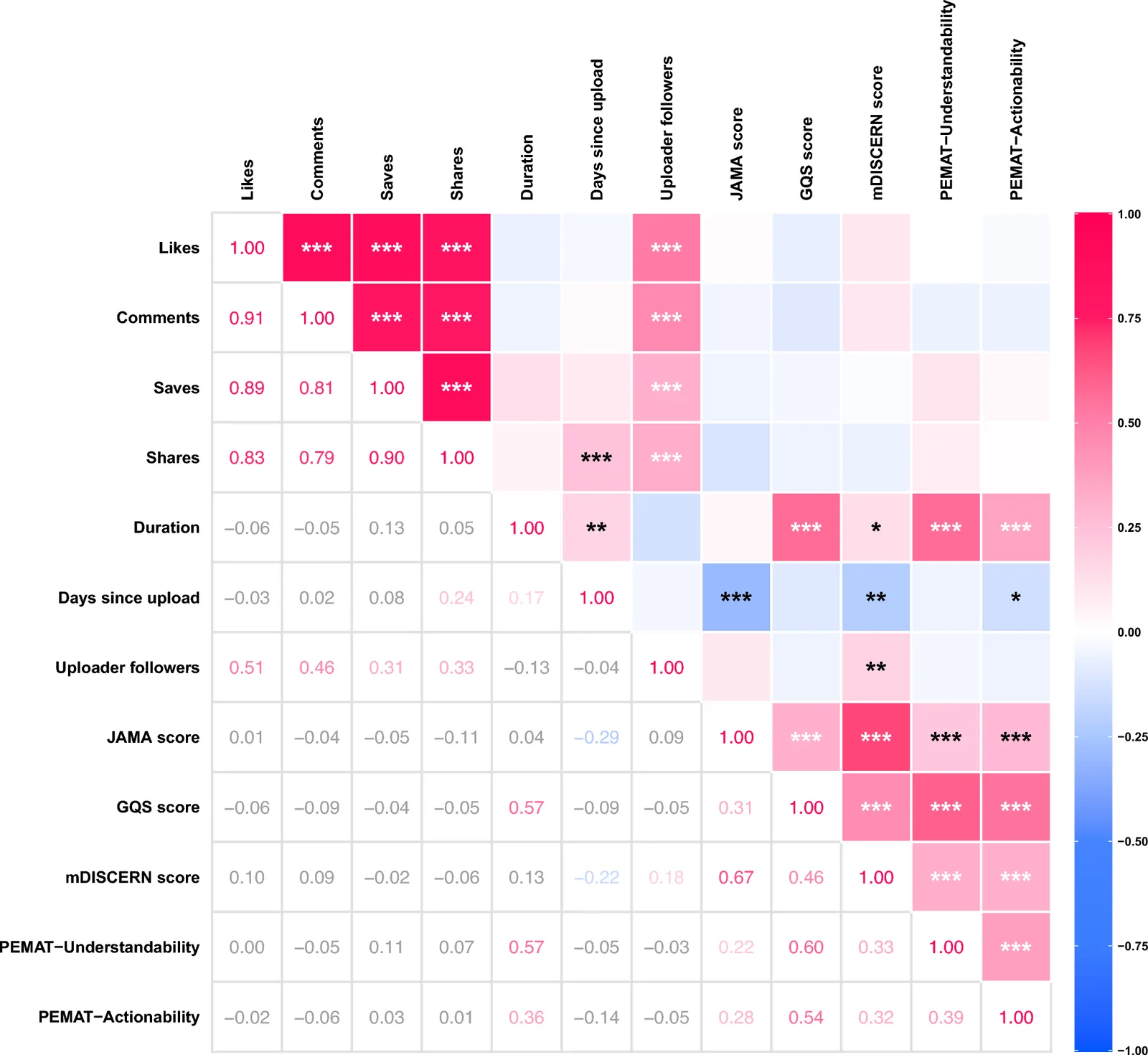
Correlation matrix heatmap of variables, encompassing visible engagement metrics (likes, comments, saves, and shares), video-specific attributes (duration, days since upload, and uploader followers), and multiple evaluative scores (JAMA score, GQS score, mDISCERN score, PEMAT-understandability, and PEMAT-actionability). The lower triangle displays Spearman’s correlation coefficients (*r*), the upper triangle indicates statistical significance, and the diagonal shows self-correlations of 1.00. The color gradient reflects the direction and strength of the correlation, with warm hues indicating positive correlations, cool hues indicating negative correlations, and darker shades indicating larger absolute correlation coefficients. * *P* < 0.05, ** *P* < 0.01, *** *P* < 0.001. JAMA, Journal of the American Medical Association; GQS, Global Quality Scale; mDISCERN, modified DISCERN; PEMAT, Patient Education Materials Assessment Tool.

All statistical analyses were primarily performed using SPSS 26.0 (IBM Corp, Armonk, NY) and GraphPad Prism 10.6 (Dotmatics, Bristol, UK), while complex correlation matrix visualizations were implemented in the R environment (Version 4.5.0) using the ‘ggplot2’ package. Based on the results of the Shapiro–Wilk normality test, continuous variables were described as Mean ± Standard Deviation (SD) or Median (Interquartile Range, IQR), whereas categorical variables were reported as frequencies (*n*) and percentages (%). Potential outliers were retained in the analysis to reflect the actual data distribution, and robust non-parametric methods were prioritized. A stratified strategy was followed for inter-group comparisons: (i) comparisons between two groups were performed using the Student’s t-test or Mann–Whitney U test; (ii) for overall differences among the three platforms, One–way ANOVA with Bonferroni post–hoc testing was applied if data met normality requirements; (iii) for non-normal data, the Kruskal–Wallis H test was primarily used for multi-group comparisons, with pairwise post–hoc comparisons adjusted via the Dunn–Bonferroni method to control the family-wise error rate. Inter-rater reliability was quantified via the Intraclass Correlation Coefficient (ICC) based on a two-way random-effects model with absolute agreement. Furthermore, monotonic correlations between variables were assessed using Spearman’s rank correlation coefficient (*r*). For overall group comparisons reported in the tables, exact *P* values are presented unless *P* < 0.001. Post hoc pairwise comparisons reported in the main text and Figs. 2, 3, 4, 5 are summarized using Dunn–Bonferroni-adjusted *P* value thresholds, with significance symbols defined in the corresponding figure legends. Statistical significance in the correlation matrix shown in Fig. 6 is indicated using *P* value thresholds defined in the figure legend. A two-sided *P* < 0.05 was considered statistically significant.

## Results

### Basic characteristics of videos

As illustrated in Fig. 1, the final analysis included 228 COPD-related short-form videos from Douyin (*n* = 86), Kwai (*n* = 92), and Bilibili (*n* = 50). Significant differences in video metrics and quality scores were observed across the platforms (Table 1). Douyin had the highest JAMA score (1.76 ± 0.53), GQS score (2.37 ± 0.77), and mDISCERN score (1.80 ± 0.79) among the three platforms (*P* < 0.001), while its video duration was at an intermediate level (median: 84 s; Q1, Q3: 48, 152).

**Table 1:** General characteristics and quality scores of COPD-related videos.

Variables	Douyin (*n* = 86)	Kwai (*n* = 92)	Bilibili (*n* = 50)	*P* value
Video metrics				
Duration, median (Q1, Q3)	84 (48, 152)	66 (31, 95)	256 (147, 413)	< 0.001
Likes, median (Q1, Q3)	371 (115, 1388)	509 (194, 2210)	69 (19, 523)	< 0.001
Comments, median (Q1, Q3)	17 (4, 77)	43 (16, 193)	4 (1, 40)	< 0.001
Saves, median (Q1, Q3)	119 (46, 472)	195 (64, 771)	99 (30, 653)	0.285
Shares, median (Q1, Q3)	81 (21, 315)	273 (81, 923)	101 (23, 391)	< 0.001
Days since upload, median (Q1, Q3)	215 (113, 415)	387 (144, 602)	885 (504, 1757)	< 0.001
Uploader followers, median (Q1, Q3)	96,000 (24,250, 313,750)	231,500 (54,000, 317,000)	13,203 (290, 90,000)	< 0.001
Quality assessment				
JAMA score, mean ± SD	1.76 ± 0.53	1.51 ± 0.54	1.30 ± 0.54	< 0.001
GQS score, mean ± SD	2.37 ± 0.77	1.90 ± 0.65	2.32 ± 0.74	< 0.001
mDISCERN score, mean ± SD	1.80 ± 0.79	1.51 ± 0.73	1.30 ± 0.79	< 0.001
PEMAT-Understandability (%), mean ± SD	83.7 ± 12.4	76.1 ± 19.9	86.3 ± 15.6	< 0.001
PEMAT-Actionability (%), mean ± SD	62.7 ± 39.8	45.3 ± 38.2	50.9 ± 46.0	0.008

Data are presented as median (Q1, Q3) for video characteristics and engagement metrics, and as mean ± SD for quality scores. Between-platform comparisons were performed using the Kruskal–Wallis H test. Exact *P* values are reported unless *P* < 0.001. *JAMA* Journal of the American Medical Association, *GQS* Global quality scale, *mDISCERN* Modified DISCERN, *PEMAT* Patient education materials assessment tool, *SD* standard deviation.

Kwai had shorter video duration and higher engagement metrics than the other platforms. This platform had the shortest video duration (median: 66 s; Q1, Q3: 31, 95) and the highest values for likes (median: 509; Q1, Q3: 194, 2210), comments (median: 43; Q1, Q3: 16, 193), and shares (median: 273; Q1, Q3: 81, 923). Although the median number of saves was numerically higher on Kwai, the difference across platforms was not statistically significant (*P* = 0.285). Furthermore, Kwai uploaders had the highest number of followers (Median: 231,500; Q1, Q3: 54,000, 317,000). However, Kwai had the lowest GQS score (1.90 ± 0.65), PEMAT-understandability score (76.1 ± 19.9%), and PEMAT-actionability score (45.3 ± 38.2%) among the three platforms.

Bilibili had the longest video duration (median: 256 s; Q1, Q3: 147, 413) and the longest time since upload (median: 885 days; Q1, Q3: 504, 1757). Although its engagement metrics, such as likes (median: 69) and comments (median: 4), were relatively low and the JAMA score (1.30 ± 0.54) was the lowest, Bilibili achieved the highest score in PEMAT-understandability (86.3 ± 15.6%).

### Video sources and content characteristics

Creator identity, content theme, presentation format, and verification status differed significantly across platforms (Table 2, *P* < 0.001). Compared with Kwai and Bilibili, Douyin had the highest proportions of videos posted by medical professionals (90.7%) and individual verified accounts (Yellow V, 91.9%). Oral monologue was the most common presentation format on Douyin (68.6%).

**Table 2:** Sources and content characteristics of COPD-related videos.

Characteristic	Douyin (*n* = 86)	Kwai (*n* = 92)	Bilibili (*n* = 50)	*P* value
Creator identity, *n* (%)				** < 0.001**
Medical professionals	78 (90.7%)	79 (85.9%)	3 (6%)	
Healthcare organizations	6 (7%)	4 (4.3%)	0 (0%)	
Independent users	2 (2.3%)	9 (9.8%)	47 (94%)	
Content theme, *n* (%)				** < 0.001**
Disease knowledge	57 (66.3%)	55 (59.8%)	47 (94%)	
Clinical scenarios	15 (17.4%)	10 (10.9%)	0 (0%)	
Intervention strategies	14 (16.3%)	27 (29.3%)	3 (6%)	
Presentation format, *n* (%)				** < 0.001**
Oral monologue	59 (68.6%)	61 (66.3%)	1 (2%)	
Visual illustration	5 (5.8%)	16 (17.4%)	38 (76%)	
Situational enactment	22 (25.6%)	15 (16.3%)	11 (22%)	
Verification status, *n* (%)				** < 0.001**
Individual verified (Yellow V)	79 (91.9%)	54 (58.7%)	6 (12%)	
Organization verified (Blue V)	5 (5.8%)	2 (2.2%)	2 (4%)	
Unverified accounts	2 (2.3%)	36 (39.1%)	42 (84%)	

Data are presented as n (%). Between-platform comparisons were performed using Fisher’s exact test with Monte Carlo simulation based on 10,000 replicates to account for small expected cell counts. Exact *P* values are reported unless P < 0.001.

Kwai also had high proportions of videos posted by medical professionals (85.9%) and oral monologue videos (66.3%). Regarding the Content theme, disease knowledge was the most common content theme on Kwai (59.8%), followed by intervention strategies (29.3%) and clinical scenarios (10.9%). In contrast, Bilibili differed from Douyin and Kwai in creator identity and verification status; most videos were posted by independent users (94.0%), and most accounts were unverified (84.0%). Visual illustration was the most common presentation format on Bilibili (76.0%). Regarding content themes, disease knowledge predominated on Bilibili (94.0%), whereas clinical scenarios and intervention strategies accounted for smaller proportions.

### Quality scores by creator identity

Quality scores differed significantly across creator identity groups (Fig. 2). Videos published by healthcare organizations had the highest scores for most quality assessment indicators; their JAMA scores (Fig. 2A) were significantly higher than those of medical professionals (*P* < 0.05) and independent users (*P* < 0.001). Healthcare organizations also had higher GQS scores (Fig. 2B) and PEMAT-understandability scores (Fig. 2D) than medical professionals (*P* < 0.05), and they had the highest PEMAT-actionability scores (Fig. 2E). Videos published by medical professionals generally showed scores between those of healthcare organizations and independent users; compared with independent users, they had higher JAMA scores (*P* < 0.001) and mDISCERN scores (Fig. 2C, *P*< 0.05). Conversely, videos produced by independent users had lower JAMA, GQS, and mDISCERN scores than videos produced by medical professionals or healthcare organizations.

### Quality scores by content theme

Quality assessment scores differed across content themes (Fig. 3). Videos categorized as clinical scenarios had higher JAMA scores (Fig. 3A) than disease knowledge videos (*P* < 0.001). Videos categorized as intervention strategies had higher GQS scores (Fig. 3B) than both clinical scenario and disease knowledge videos (*P* < 0.05), and their PEMAT-actionability scores (Fig. 3E) were higher than those of clinical scenario videos (*P* < 0.05). Disease knowledge videos had lower JAMA scores than intervention strategy videos (*P* < 0.05). No statistically significant differences were observed across content themes for mDISCERN scores (Fig. 3C) or PEMAT-understandability scores (Fig. 3D).

### Quality scores by presentation format

Quality scores differed significantly across presentation formats (Fig. 4**)**. Oral monologue videos had higher GQS scores (Fig. 4B) and PEMAT-actionability scores (Fig. 4E) than visual illustration videos (*P* < 0.01). For transparency and reliability indicators, both oral monologue and situational enactment videos had higher JAMA scores (Fig. 4A) and mDISCERN scores (Fig. 4C) than visual illustration videos (*P* < 0.001). No significant differences were observed across presentation formats for PEMAT-understandability scores (Fig. 4D).

### Quality scores by verification status

Quality scores differed significantly across verification status groups (Fig. 5). Organization verified accounts (Blue V) had the highest scores for several quality assessment indicators. Specifically, Blue V accounts had the highest JAMA scores (Fig. 5A), GQS scores (Fig. 5B), and mDISCERN scores (Fig. 5C). These scores were significantly higher than those of unverified accounts (*P* < 0.001) and individual verified accounts (Yellow V) (*P* < 0.05 or *P* < 0.01). Blue V accounts also had the highest PEMAT-understandability scores (Fig. 5D), which were significantly higher than those of the other two account types (*P* < 0.01). Individual verified accounts (Yellow V) did not show consistently higher quality scores than unverified accounts across all indicators. No significant differences were observed across verification status groups for PEMAT-actionability scores (Fig. 5E).

### Correlation analysis

Spearman correlation analysis revealed significant positive correlations among likes, comments, saves, and shares (Fig. 6). The strongest correlation was observed between likes and comments (*r* = 0.91), followed by likes and saves (*r* = 0.89) and likes and shares (*r* = 0.83). Regarding video characteristics, video duration was moderately positively correlated with both the GQS score (*r* = 0.57) and PEMAT-understandability (%) (*r* = 0.57). Within the quality assessment indicators, JAMA, GQS, mDISCERN, PEMAT-understandability, and PEMAT-actionability scores were generally positively correlated with one another. The strongest correlation among quality indicators was observed between JAMA and mDISCERN scores (*r* = 0.67), followed by the correlation between GQS and PEMAT-understandability scores (*r* = 0.60). Engagement metrics showed no significant correlation with quality scores, including JAMA scores (*r* = 0.01) and GQS scores (*r* = -0.06).

## Discussion

With the “Healthy China 2030” initiative emphasizing the prevention and management of chronic respiratory diseases, the quality of digital health information related to COPD has become an important issue in public health communication^45,46^. In this cross-sectional content analysis of 228 COPD-related videos from three Chinese short-form video platforms, we observed substantial platform-specific differences in source profiles, presentation formats, quality scores, and visible engagement. Overall, COPD-related videos varied considerably in transparency, reliability, understandability, actionability, and public visibility. These findings suggest that improving digital COPD communication requires not only the production of professionally credible content, but also clearer source identification and more accessible formats for patient education. This interpretation is consistent with previous concerns regarding the variable quality of health information on social media platforms^21,27–29^.

### Variation in video quality across platforms

The multi-platform comparison showed significant differences in video quality, source characteristics, and engagement patterns across platforms. Overall, the quality of COPD-related videos remained suboptimal, particularly in transparency and reliability, as reflected by the relatively low JAMA and mDISCERN scores. Similar concerns have been reported in studies evaluating COPD-related videos on TikTok and YouTube, which found limitations in reliability, credibility, or guideline consistency^27,31^. In the present study, Douyin had the highest JAMA and GQS scores, and most Douyin videos were posted by medical professionals or individual verified accounts. This pattern suggests that source characteristics and verification status may be associated with information quality, although the present cross-sectional design cannot determine whether platform mechanisms directly caused these differences.

Kwai and Bilibili showed higher proportions of unverified or independent-user content, which may partly reflect differences in platform communities, creator composition, and user cultures. Previous research on Kwai has described its broad and socially diverse user base, including users from lower-tier cities and rural areas, suggesting that platform-specific communities may influence the types and presentation styles of health content available to audiences^47^. Previous reviews of health misinformation on social media have also emphasized that heterogeneous user-generated information environments can complicate the public’s ability to identify reliable medical information and may create challenges for clinical communication^25,48^. These findings support the need for continued cross-platform monitoring of COPD-related health information, rather than relying on platform popularity or engagement metrics alone.

### Video quality and visible engagement

This study observed a weak association between visible engagement metrics and medical quality indicators. Although likes, comments, saves, and shares were strongly correlated with one another, these engagement metrics were not significantly correlated with JAMA or GQS scores. Visible popularity should therefore not be interpreted as a proxy for medical reliability or educational quality.

This observation is consistent with recent studies of health information on short-form video and video-sharing platforms. In a cross-platform analysis of lung cancer information on TikTok, Kwai, and Rednote, Zheng et al. reported substantial heterogeneity in the quality, reliability, and dissemination patterns of health-related short-form videos^49^. Together with previous studies of COPD-related videos on YouTube and TikTok, the present findings suggest that the mismatch between visible engagement and quality may represent a broader challenge in digital health communication, rather than a problem limited to a single disease or platform^27,31^.

Several factors may contribute to the weak association between engagement and quality, including creator identity, content topic, presentation style, platform environment, uploader follower count, user preferences, and possibly recommendation mechanisms. However, this study did not directly measure algorithmic exposure, impressions, watch time, ranking changes, or longitudinal recommendation pathways. Algorithmic recommendation should therefore be interpreted only as a possible explanatory factor, not as a mechanism demonstrated by the present data.

From a communication perspective, users may respond to short-form videos through peripheral cues such as visual salience, emotional tone, narrative structure, or perceived source identity, rather than through systematic evaluation of medical evidence. Viewed through the Elaboration Likelihood Model^50^, these peripheral cues may help explain why visible engagement was weakly aligned with medical quality. This interpretation remains exploratory because viewer cognition and emotional responses were not directly measured. Future studies should combine content analysis with longitudinal platform audits, exposure data, recommendation-pathway tracking, and user-level behavioral data to clarify how high-quality COPD information is distributed and consumed on short-form video platforms.

### Presentation formats and information comprehension

Reducing cognitive load is important for effective COPD education, particularly because many patients have difficulty understanding disease mechanisms and self-management requirements. In the present study, Bilibili had the highest PEMAT-understandability score and the longest video duration. The moderate correlation between duration and PEMAT-understandability suggests that longer videos may provide more space for explanation. Visual illustration may also help translate complex medical information into more accessible visual representations, which is consistent with evidence supporting video-based and visual health education approaches^51,52^. Accordingly, understandability should be interpreted as a communication attribute rather than a proxy for transparency, reliability, or clinical accuracy.

### Content themes and quality-related risks

Because intervention-related videos may directly influence self-management behaviors, their quality warrants particular attention. In this study, intervention strategies accounted for a relatively high proportion of Kwai videos, whereas Kwai also showed lower overall quality scores. This pattern should be interpreted cautiously because the analysis did not determine whether content theme contributed to platform-level quality differences, and guideline concordance and specific misinformation types were not directly coded. Nevertheless, previous research has shown that health misinformation and unsupported claims are common on social media, particularly in areas involving treatment, lifestyle modification, and commercialized health advice^25,26,53^. A previous YouTube-based study on COPD treatment also found substantial limitations in the guideline consistency and reliability of online COPD treatment information^31^. Public health authorities and platforms should therefore consider prioritizing the monitoring of high-risk therapeutic claims, especially those related to pharmacological treatment, inhaler use, breathing exercises, rehabilitation, and self-management.

### Creator identity and presentation format

Creator identity and presentation format were both associated with differences in quality scores. Videos from healthcare organizations generally achieved higher quality scores, whereas videos from independent users had lower scores for several indicators. However, professional identity alone does not guarantee effective communication. For COPD education, particularly inhaler use, breathing exercises, and rehabilitation, practical demonstrations may be especially important because they translate abstract recommendations into observable behaviors. This interpretation is consistent with evidence suggesting that video-based health education can improve information delivery and support patient understanding of health behaviors^54^. Future content creation should therefore combine medical accuracy with accessible formats, including visual demonstration, structured explanation, and patient-centered language.

### Verification mechanisms and information reliability

Verification status was associated with differences in quality scores, with organization verified accounts generally showing higher scores. This finding suggests that verification markers may help users identify potentially more reliable sources, although verification status alone should not be treated as a guarantee of medical accuracy. Similar findings have been reported in previous studies of short-form video health information, in which professionally affiliated or institutionally verified sources tended to show higher reliability or quality scores^33,55^. Future platform governance may benefit from combining identity verification with clearer health-information labeling and content quality review, particularly for therapeutic or self-management content.

At the governance level, recent policy discussions, including the EU Digital Services Act and broader interventions addressing misleading medical information online, highlight the need for more transparent and accountable management of digital information environments^56,57^. However, the effectiveness of specific recommendation, labeling, or ranking interventions should be evaluated in future platform-level studies.

### Limitations

This study has several limitations. First, as a cross-sectional content analysis, the data were collected within a consecutive 48-h window from December 26 to 28, 2025. The study therefore could not capture longitudinal changes in search ranking, algorithmic exposure, platform recommendation pathways, or visible engagement over time. Second, the search strategy used only the standard Chinese medical term “慢性阻塞性肺疾病” (chronic obstructive pulmonary disease). Although this approach ensured medical precision, it may have excluded relevant videos using colloquial expressions, abbreviations, or lay terms commonly used by patients. Third, the analysis was restricted to the top 100 videos retrieved from each platform. This strategy approximated typical user-facing search results but did not capture the long-tail distribution of COPD-related content.

Fourth, engagement indicators, including likes, comments, saves, and shares, were cumulative platform-displayed metrics. These indicators may have been influenced by days since upload, uploader follower count, platform traffic, topic salience, and unmeasured algorithmic exposure. Therefore, comparisons of engagement across platforms should be interpreted cautiously. Fifth, videos published within seven days were excluded to reduce instability in early engagement metrics; however, this criterion may have omitted rapidly trending videos, including newly emerging health misinformation or time-sensitive health claims. Finally, this study was limited to Chinese-language videos on three Chinese platforms, which may restrict the generalizability of the findings to other languages, platforms, or regulatory environments. Future studies should use longitudinal sampling, repeated searches, multi-keyword strategies, and platform-level exposure data to characterize dynamic changes in ranking, engagement, and the dissemination of COPD-related health information.

### Recommendations

Based on these findings, several practical strategies may help improve COPD-related digital health communication. First, healthcare professionals could use information prescriptions. Clinicians and healthcare institutions could proactively recommend professionally verified or evidence-based online health information sources to help patients identify reliable COPD-related information without relying on visible popularity as a proxy for quality. Second, healthcare institutions and content creators could develop doctor-patient-media co-creation models. Such collaborations could produce standardized, patient-centered videos that combine clinical accuracy with accessible formats, including visual demonstrations, structured explanations, and plain-language communication. Third, platform administrators and policymakers could strengthen platform governance and content labeling. Platforms could consider clearer health-information labels, greater visibility for verified medical or institutional sources, and targeted review of high-risk therapeutic claims. These claims may include content involving pharmacological treatment, inhaler use, breathing exercises, rehabilitation, and self-management. Because this study did not directly evaluate platform-level interventions, future longitudinal or experimental studies are needed to assess the effectiveness of ranking, labeling, or recommendation-based strategies.

## Conclusions

This cross-sectional content analysis showed substantial variation in the quality, reliability, understandability, actionability, and engagement characteristics of COPD-related videos across three major Chinese short-form video platforms. Overall, transparency and reliability were suboptimal, and visible engagement metrics were not significantly correlated with medical quality indicators. Videos from professional or institutionally verified sources tended to achieve higher quality scores, suggesting that source characteristics and verification status remain important for COPD-related digital health communication. These findings indicate that healthcare professionals should guide patients toward credible online information sources and develop more accessible evidence-based video content, while policymakers and platform administrators should strengthen source identification, health-information labeling, and oversight of high-risk therapeutic claims. Because this study did not measure algorithmic exposure, impressions, watch time, or longitudinal ranking changes, the observed quality–engagement mismatch should be interpreted as an association rather than evidence that platform algorithms directly caused it.

## Supplementary Information

Supplementary Information.

## Data Availability

All videos analyzed in this study were publicly available at the time of data collection. The study did not involve access to private or restricted data. The search strategy, inclusion criteria, and evaluation methods are described in detail in the Methods section to enable reproducibility. The original video content remains accessible via the following public platforms: Douyin (https://www.douyin.com), Kwai (https://www.kuaishou.com), and Bilibili (https://www.bilibili.com). The de-identified coded dataset supporting the findings of this study is available from the corresponding author upon reasonable academic request. Original video URLs were retained internally for verification and traceability and may be shared for academic verification where permitted by platform terms and applicable ethical requirements.

## Abbreviations

COPD: Chronic obstructive pulmonary disease
ICC: Intraclass correlation coefficient
JAMA: Journal of the American Medical Association
GQS: Global quality scale
PEMAT: Patient education materials assessment tool
mDISCERN: Modified DISCERN

## References

[1] 2026 GOLD report and pocket guide. *Global Initiative for Chronic Obstructive Lung Disease.* https://goldcopd.org/2026-gold-report-and-pocket-guide/ (2026).

[2] The silent killer: why chronic respiratory disease deserves global attention. *World Health Organization.* https://www.who.int/news-room/commentaries/detail/the-silent-killer--why-chronic-respiratory-disease-deserves-global-attention (2024).

[3] Adeloye, D. et al. Global, regional, and national prevalence of, and risk factors for, chronic obstructive pulmonary disease (COPD) in 2019: a systematic review and modelling analysis. *Lancet Respir. Med.* **10**, 447–458 (2022). 35279265 10.1016/S2213-2600(21)00511-7 PMC9050565

[4] Wang, C. et al. Prevalence and risk factors of chronic obstructive pulmonary disease in China (the China pulmonary health [CPH] study): a national cross-sectional study. *Lancet* **391**, 1706–1717 (2018). 29650248 10.1016/S0140-6736(18)30841-9

[5] Chen, S. et al. The global economic burden of chronic obstructive pulmonary disease for 204 countries and territories in 2020–50: a health-augmented macroeconomic modelling study. *Lancet Glob. Health* **11**, e1183–e1193 (2023). 37474226 10.1016/S2214-109X(23)00217-6 PMC10369014

[6] Hopkinson, N. S. et al. Early life exposures and the development of chronic obstructive pulmonary disease across the life course. *Am. J. Respir. Crit. Care Med.* **210**, 572–580 (2024). 38861321 10.1164/rccm.202402-0432PP

[7] Marcon, A. et al. The coexistence of asthma and COPD: risk factors, clinical history and lung function trajectories. *Eur. Respir. J.* **58**, 2004656 (2021). 33863744 10.1183/13993003.04656-2020 PMC8613837

[8] Schrijver, J. et al. Self-management interventions for people with chronic obstructive pulmonary disease. *Cochrane Database Syst. Rev.* **2022**, CD002990 (2022). 10.1002/14651858.CD002990.pub4 PMC8743569 35001366

[9] Jeganathan, C. & Hosseinzadeh, H. The role of health literacy on the self-management of chronic obstructive pulmonary disease: a systematic review. *COPD* **17**, 318–325 (2020). 32506965 10.1080/15412555.2020.1772739

[10] Gogou, E. et al. Underestimation of respiratory symptoms by smokers: a thorn in chronic obstructive pulmonary disease diagnosis. *npj Primary Care Respir. Med.* **31**, 14 (2021). 33712602 10.1038/s41533-021-00226-y PMC7955112

[11] Xia, Y., Zhang, Y. & Su, D. Knowledge, attitudes, and practices of COPD patients regarding acute exacerbations of COPD. *Front. Med.* **12**, 1620703 (2025). 10.3389/fmed.2025.1620703 PMC12440717 40969791

[12] Bischof, A. Y., Cordier, J., Vogel, J. & Geissler, A. Medication adherence halves COPD patients’ hospitalization risk: evidence from Swiss health insurance data. *npj Primary Care Respir. Med.* **34**, 1 (2024). 10.1038/s41533-024-00361-2 PMC10920735 38453930

[13] Yi, S., Guo, Y., Lin, Z. & Cao, C. Is information evaluated subjectively? Social media has changed the way users search for medical information. *Dig. Health ***10**, 20552076241259039 (2024). 10.1177/20552076241259039 PMC11135116 38812844

[14] The 56th Statistical Report on the Development Status of China’s Internet -- Research on Internet Development. *China Internet Network Information Center.* https://www.cnnic.org.cn/n4/2025/0721/c88-11328.html (2025).

[15] Hu, Y. et al. How to select high-quality health educational short videos on social media? Insights from YouTube and Douyin. *BMC Med. Educ.* **25**, 1418 (2025). 41088163 10.1186/s12909-025-08018-5 PMC12523207

[16] Galmarini, E., Marciano, L. & Schulz, P. J. The effectiveness of visual-based interventions on health literacy in health care: a systematic review and meta-analysis. *BMC Health Serv. Res.* **24**, 718 (2024). 38862966 10.1186/s12913-024-11138-1 PMC11165863

[17] Deshpande, N. et al. Video-based educational interventions for patients with chronic illnesses: systematic review. *J. Med. Internet Res.* **25**, e41092 (2023). 37467015 10.2196/41092 PMC10398560

[18] Su, L. et al. Short video platforms as sources of health information about cervical cancer screening: a content and quality analysis. *Dig. Health* **11**, 20552076251404516 (2025). 10.1177/20552076251404516 PMC12673052 41346941

[19] Su, L. et al. Short video platforms as sources of health information about HPV vaccine: a content and quality analysis. *Dig. Health* **11**, 20552076251379340 (2025). 10.1177/20552076251379340 PMC12437182 40964602

[20] He, F. et al. Quality and reliability of pediatric pneumonia related short videos on mainstream platforms: cross-sectional study. *BMC Public Health* **25**, 1896 (2025). 40410758 10.1186/s12889-025-22963-2 PMC12101000

[21] Zeng, F. et al. Douyin and Bilibili as sources of information on lung cancer in China through assessment and analysis of the content and quality. *Sci. Rep.* **14**, 20604 (2024). 39232044 10.1038/s41598-024-70640-y PMC11375008

[22] Lei, Y., Liao, F., Li, X. & Zhu, Y. Quality and reliability evaluation of pancreatic cancer-related video content on social short video platforms: a cross-sectional study. *BMC Public Health* **25**, 1919 (2025). 40413453 10.1186/s12889-025-23130-3 PMC12102813

[23] Ji, G. Kuaishou short video social network from the perspective of urban-rural cultural linkage: a field study on the mobile internet practices of young Monguor villagers in China’s Qinghai province. *Int. J. Anthropol. Ethnol.* **5**, 8 (2021).

[24] Wang, X., Wuji, S., Liu, Y., Luo, R. & Qiu, C. Study on the impact of recommendation algorithms on user perceived stress and health management behaviour in short video platforms. *Inf. Process. Manage.* **61**, 103674 (2024).

[25] Suarez-Lledo, V. & Alvarez-Galvez, J. Prevalence of health misinformation on social media: systematic review. *J. Med. Internet Res.* **23**, e17187 (2021). 33470931 10.2196/17187 PMC7857950

[26] Ricke, J.-N. & Seifert, R. Disinformation on dietary supplements by German influencers on Instagram. *Naunyn-Schmiedeberg’s Arch. Pharmacol.* **398**, 5629–5647 (2025). 39585397 10.1007/s00210-024-03616-4 PMC11985574

[27] Song, S. et al. Short-video apps as a health information source for chronic obstructive pulmonary disease: information quality assessment of TikTok videos. *J. Med. Internet Res.* **23**, e28318 (2021). 34931996 10.2196/28318 PMC8726035

[28] Sun, K.-X. et al. Reliability, quality, and science popularization of diabetes knowledge information in China video sharing platform: a cross-sectional study. *Dig. Health* **11**, 20552076251393298 (2025). 10.1177/20552076251393298 PMC12576113 41181547

[29] Li, J. et al. Quality and reliability of Alzheimer’s disease videos on Douyin and Bilibili: a cross-sectional content analysis study. *Dig. Health* **11**, 20552076251398464 (2025). 10.1177/20552076251398464 PMC12638718 41278376

[30] Murray, J., McNally, E. & Kent, B. P56 clinical accuracy and risk of harm in asthma related content on TikTok. *Thorax* **78**, A139–A139 (2023). 10.1016/j.chest.2024.09.047 39528109

[31] Rodrigues, A. L. et al. Analysis of content, credibility, and reliability of information on chronic obstructive pulmonary disease treatment in YouTube^TM^ videos: a cross-sectional observational study. *Fisioter. Pesqui.* **32**, e23017924en (2025).

[32] Peng, J. et al. Assessment of the reliability and quality of pancreatic cancer related short videos on mainstream platforms: cross-sectional study. *BMC Cancer* **25**, 1428 (2025). 40993566 10.1186/s12885-025-14825-2 PMC12461966

[33] Zhang, Y. et al. Quality assessment of spinal cord injury-related health information on short-form video platforms: cross-sectional content analysis of TikTok, kwai, and BiliBili. *Dig. Health* **11**, 20552076251374226 (2025). 10.1177/20552076251374226 PMC12409039 40918080

[34] Permenov, B. A. et al. Evaluating the quality and reliability of YouTube as a source of information on extracorporeal membrane oxygenation: a call to publish more quality videos by professionals. *J. Korean Med. Sci.* **40**, e34 (2024). 10.3346/jkms.2025.40.e34 PMC11976102 40195923

[35] Karataş, L., Utkan Karasu, A. & Demirsoy, N. Is YouTube a sufficient and reliable source to inform patients about cardiac rehabilitation?: a cross-sectional study. *J. Cardiopulm. Rehabil. Prev.* **44**, 239–247 (2024). 10.1097/HCR.0000000000000864 38875164

[36] Yemeshev, Y., Bekaryssova, D. & Kocyigit, B. F. Assessment of the quality and reliability of YouTube videos related to teleradiology in musculoskeletal and rheumatic diseases: a cross-sectional study. *Rheumatol. Int.* **45**, 74 (2025). 40085231 10.1007/s00296-025-05831-5 PMC11909036

[37] Silberg, W. M., Lundberg, G. D. & Musacchio, R. A. Assessing, controlling, and assuring the quality of medical information on the internet: caveant lector et viewor-let the reader and viewer beware. *JAMA* **277**, 1244–1245 (1997). 9103351

[38] Oremule, B., Patel, A., Orekoya, O., Advani, R. & Bondin, D. Quality and reliability of YouTube videos as a source of patient information on rhinoplasty. *JAMA Otolaryngol. Head Neck Surg.* **145**, 282–283 (2019). 30605207 10.1001/jamaoto.2018.3723 PMC6440240

[39] Sun, F., Zheng, S. & Wu, J. Quality of information in gallstone disease videos on TikTok: cross-sectional study. *J. Med. Internet Res.* **25**, e39162 (2023). 36753307 10.2196/39162 PMC9947761

[40] Guler, M. A. & Aydın, E. O. Development and validation of a tool for evaluating YouTube-based medical videos. *Ir. J. Med. Sci.* **191**, 1985–1990 (2022). 34825344 10.1007/s11845-021-02864-0 PMC8616030

[41] He, Z. et al. The reliability and quality of short videos as a source of dietary guidance for inflammatory bowel disease: cross-sectional study. *J. Med. Internet Res.* **25**, e41518 (2023). 36757757 10.2196/41518 PMC9951074

[42] Shoemaker, S. J., Wolf, M. S. & Brach, C. Development of the patient education materials assessment tool (PEMAT): a new measure of understandability and actionability for print and audiovisual patient information. *Patient Educ. Couns.* **96**, 395–403 (2014). 24973195 10.1016/j.pec.2014.05.027 PMC5085258

[43] Vishnevetsky, J., Walters, C. B. & Tan, K. S. Interrater reliability of the patient education materials assessment tool (PEMAT). *Patient Educ. Couns.* **101**, 490–496 (2018). 28899713 10.1016/j.pec.2017.09.003 PMC5839932

[44] Koo, T. K. & Li, M. Y. A guideline of selecting and reporting intraclass correlation coefficients for reliability research. *J. Chiropr. Med.* **15**, 155–163 (2016). 27330520 10.1016/j.jcm.2016.02.012 PMC4913118

[45] Interpretation of the Implementation Plan for Chronic Respiratory Disease Prevention and Control Action under the Healthy China Initiative (2024–2030). *National Health Commission of the People’s Republic of China.* https://www.nhc.gov.cn/ylyjs/gzdt/202407/a0f34e52a6404ec4b4141e3fe4a85338.shtml (2024).

[46] Inclusion of Chronic Obstructive Pulmonary Disease in China’s National Basic Public Health Service Program. *National Center for Chronic and Noncommunicable Disease Control and Prevention, China CDC.* https://ncncd.chinacdc.cn/zxdt/202410/t20241015_301519.htm (2024).

[47] Tan, C. K., Wang, J., Wangzhu, S., Xu, J. & Zhu, C. The real digital housewives of China’s Kuaishou video-sharing and live-streaming app. *Media Cult. Soc.* **42**, 1243–1259 (2020).

[48] Kbaier, D., Kane, A., McJury, M. & Kenny, I. Prevalence of health misinformation on social media—challenges and mitigation before, during, and beyond the COVID-19 pandemic: scoping literature review. *J. Med. Internet Res.* **26**, e38786 (2024). 39159456 10.2196/38786 PMC11369541

[49] Zheng, X., Li, Q., Jin, L., Shi, K. & Deng, M. Quality, reliability, and dissemination of lung cancer information on short-video platforms in China: a cross-platform content analysis of TikTok, Kwai, and Rednote. *Front. Public Health* **13**, 1683561 (2025). 41404572 10.3389/fpubh.2025.1683561 PMC12702867

[50] Petty, R. E. & Cacioppo, J. T. The elaboration likelihood model of persuasion. In *Advances in Experimental Social Psychology* Vol. 19 123–205 (Academic Press, 1986).

[51] Frick, P., Kendeou, P. & Schüler, A. Knowledge revision processes during reading: how pictures influence the activation of outdated information. *Mem. Cognit.* **53**, 547–567 (2025). 38831159 10.3758/s13421-024-01586-9 PMC11868230

[52] Silliman, M. et al. Comparing lengths and inclusion of information in storytelling videos: implications for hepatitis B education. *PEC Innov.* **1**, 100049 (2022). 37213761 10.1016/j.pecinn.2022.100049 PMC10194295

[53] Nguyen, V. et al. Unravelling the truth: examining the evidence for health-related claims made by naturopathic influencers on social media – a retrospective analysis. *Health Promot. Perspect.* **12**, 372–380 (2022). 36852198 10.34172/hpp.2022.49 PMC9958238

[54] Morgado, M. et al. Video-based approaches in health education: a systematic review and meta-analysis. *Sci. Rep.* **14**, 23651 (2024). 39384592 10.1038/s41598-024-73671-7 PMC11464794

[55] Guo, F., Ding, G., Zhang, Y. & Liu, X. Quality assessment of radiotherapy health information on short-form video platforms of TikTok and bilibili: cross-sectional study. *JMIR Cancer* **11**, e73455 (2025). 40986789 10.2196/73455 PMC12456845

[56] Gram, E. G. et al. Addressing misleading medical information on social media: a scoping review of current interventions. *BMJ Evid. Based Med.* **30**, 420–433 (2025). 41047164 10.1136/bmjebm-2025-113704 PMC12703284

[57] Regulation (EU) 2022/2065 of the European Parliament and of the Council of 19 October 2022 on a Single Market for Digital Services and Amending Directive 2000/31/EC (Digital Services Act). *Off. J. Eur. Union L* **277** (2022).

