# Supplementary material for: Quality, reliability, and engagement of COPD-related videos on Chinese short-form video platforms: a cross-sectional content analysis
Source: Sci Rep. 2026 Jun 18;16:27883. doi: 10.1038/s41598-026-57615-x (PMC13547415; doi:10.1038/s41598-026-57615-x)
Supplement: Supplementary file 1 [file 41598_2026_57615_MOESM1_ESM.pdf]

Table S1. STROBE Statement—Checklist of items that should be included in reports of ***cross-sectional studies***

	Item No	Recommendation	Page No
**Title and abstract**	1	(*a*) Indicate the study's design with a commonly used term in the title or the abstract	P1 (Title)
		(*b*) Provide in the abstract an informative and balanced summary of what was done and what was found	P2 (Abstract)
**Introduction**			
Background/rationale	2	Explain the scientific background and rationale for the investigation being reported	P2-P4 (Introduction)
Objectives	3	State specific objectives, including any prespecified hypotheses	P4 (Introduction)
**Methods**			
Study design	4	Present key elements of study design early in the paper	P4-P5 (Methods: Study Design)
Setting	5	Describe the setting, locations, and relevant dates, including periods of recruitment, exposure, follow-up, and data collection	P5 (Methods: Search Strategy and Data Collection)
Participants	6	(*a*) Give the eligibility criteria, and the sources and methods of selection of participants	P5-P6 (Methods: Inclusion/Exclusion Criteria)
Variables	7	Clearly define all outcomes, exposures, predictors, potential confounders, and effect modifiers. Give diagnostic criteria, if applicable	P6-P8 (Methods: Data Extraction; Video Content and Quality Assessment)
Data sources/ measurement	8*	For each variable of interest, give sources of data and details of methods of assessment (measurement). Describe comparability of assessment methods if there is more than one group	P5-P8 (Methods: Search Strategy and Data Collection; Data Extraction; Video Content and Quality Assessment)
Bias	9	Describe any efforts to address potential sources of bias	P5-P6 (Search/Exclusion Criteria); P8-P9 (Inter-rater Reliability)
Study size	10	Explain how the study size was arrived at	P5-P6 (Methods: Search Strategy and Data Collection; Data Extraction)
Quantitative variables	11	Explain how quantitative variables were handled in the analyses. If applicable, describe which groupings were chosen and why	P6-P8 (Methods: Data Extraction; Video Content and Quality Assessment); P9–P10 (Statistical Analysis)
Statistical methods	12	(*a*) Describe all statistical methods, including those used to control for confounding	P9–P10 (Methods: Statistical Analysis)

		(*b*) Describe any methods used to examine subgroups and interactions	P10 (Statistical Analysis)
		(*c*) Explain how missing data were addressed	P6 (Exclusion Criteria - unstable data)
		(*d*) If applicable, describe analytical methods taking account of sampling strategy	P5-P6 (Search Strategy and Data Collection; Data Extraction); P10 (Statistical Analysis)
		(*e*) Describe any sensitivity analyses	N/A (Not explicitly stated, primarily descriptive/comparative)
**Results**			
Participants	13*	(a) Report numbers of individuals at each stage of study—eg numbers potentially eligible, examined for eligibility, confirmed eligible, included in the study, completing follow-up, and analysed	P6 (Data Extraction); P11 (Results); Fig. 1 legend on P28
		(b) Give reasons for non-participation at each stage	P6 (Data Extraction); Fig. 1 legend on P28
		(c) Consider use of a flow diagram	Fig. 1; legend on P28
Descriptive data	14*	(a) Give characteristics of study participants (eg demographic, clinical, social) and information on exposures and potential confounders	P11-P13 (Results); P30 (Table 1); P31 (Table 2)
		(b) Indicate number of participants with missing data for each variable of interest	N/A (Samples with unstable data were excluded)
Outcome data	15*	Report numbers of outcome events or summary measures	P11-P14 (Results); P30 (Table 1); P31 (Table 2)
Main results	16	(*a*) Give unadjusted estimates and, if applicable, confounder-adjusted estimates and their precision (eg, 95% confidence interval). Make clear which confounders were adjusted for and why they were included	P11-P14 (Results); P30-P31 (Tables 1-2); Fig. 2-Fig. 6 legends on P28-P29
		(*b*) Report category boundaries when continuous variables were categorized	P7-P8 (Methods: Data Extraction; Video Content and Quality Assessment)
		(*c*) If relevant, consider translating estimates of relative risk into absolute risk for a meaningful time period	N/A (Descriptive Cross-sectional)
Other analyses	17	Report other analyses done—eg analyses of subgroups and interactions, and sensitivity analyses	P13-P14 (Results: Correlation analysis); Fig. 6 legend on P29
**Discussion**			
Key results	18	Summarise key results with reference to study objectives	P14-P15 (Discussion)
Limitations	19	Discuss limitations of the study, taking into account sources of potential bias or imprecision. Discuss both direction and magnitude of any potential bias	P19 (Discussion: Limitations)
Interpretation	20	Give a cautious overall interpretation of results considering objectives, limitations, multiplicity of	P14-P20 (Discussion)

		analyses, results from similar studies, and other relevant evidence	
Generalisability	21	Discuss the generalisability (external validity) of the study results	P19 (Limitations)
Other information			
Funding	22	Give the source of funding and the role of the funders for the present study and, if applicable, for the original study on which the present article is based	P22 (Funding statement)

*Give information separately for exposed and unexposed groups.

Note: An Explanation and Elaboration article discusses each checklist item and gives methodological background and published examples of transparent reporting. The STROBE checklist is best used in conjunction with this article (freely available on the Web sites of PLoS Medicine at http://www.plosmedicine.org/, Annals of Internal Medicine at http://www.annals.org/, and Epidemiology at http://www.epidem.com/). Information on the STROBE Initiative is available at www.strobe-statement.org.

Table S2. The Journal of American Medical Association (JAMA) benchmark criteria.

Criteria (1 point for each)	Description
Authorship	Author and contributor credentials and their affiliations should be provided
Attribution	Clearly lists all copyright information and states references and sources for content
Currency	Initial date of posted content and subsequent updates to content should be provided
Disclosure	Conflicts of interest, funding, sponsorship, advertising, support, and video ownership should be fully disclosed

Table S3. Description of the Global Quality Score (GQS) scale.

Scale	Description
Poor quality (1 point)	Poor quality and poor flow of the site, most information missing, not at all useful for patients
Generally poor quality (2 point)	Generally poor quality and poor flow, some information listed but many important topics missing, of very limited use to patients
Moderate quality (3 point)	Moderate quality, sub-optimal flow, some important information is adequately discussed but others poorly discussed, somewhat useful for patients
Good quality (4 point)	Good quality and generally good flow, most of the relevant information is listed, but some topics not covered, useful for patients

Excellent quality (5 point)	Excellent quality and excellent flow, very useful for patients

Table S4. Description of the modified DISCERN (mDISCERN) score.

Criteria (1 point for each)	Description
1	Is the video clear, concise, and understandable?
2	Are reliable sources of information used? (i.e., publication cited, speaker is specialist)
3	Is the information presented balanced and unbiased?
4	Are additional sources of information listed for patient reference?
5	Are areas of uncertainty/controversy mentioned?

Table S5. The Patient Education Materials Assessment Tool (PEMAT) Understandability.

	Item	Response Options	Rating
Topic: Content			
1	The material makes its purpose completely evident.	Disagree=0, Agree=1	
Topic: Word Choice & Style			
3	The material uses common, everyday language.	Disagree=0, Agree=1	
4	Medical terms are used only to familiarize audience with the terms. When used, medical terms are defined.	Disagree=0, Agree=1	
5	The material uses the active voice.	Disagree=0, Agree=1	
Topic: Organization			
8	The material breaks or "chunks" information into short sections.	Disagree=0, Agree=1, Very short material=N/A	
9	The material's sections have informative headers.	Disagree=0, Agree=1, Very short material=N/A	
10	The material presents information in a logical sequence.	Disagree=0, Agree=1	
11	The material provides a summary.	Disagree=0, Agree=1, Very short material=N/A	
Topic: Layout & Design			

12	The material uses visual cues (e.g., arrows, boxes, bullets, bold, larger font, highlighting) to draw attention to key points.	Disagree=0, Agree=1, Video=N/A
13	Text on the screen is easy to read.	Disagree=0, Agree=1, No text or all text is narrated=N/A
14	The material allows the user to hear the words clearly (e.g., not too fast, not garbled).	Disagree=0, Agree=1, No narration=N/A
Topic: Use of Visual Aids		
18	The material uses illustrations and photographs that are clear and uncluttered.	Disagree=0, Agree=1, No visual aids=N/A
19	The material uses simple tables with short and clear row and column headings.	Disagree=0, Agree=1, No tables=N/A

Total Points: _____________
Total Possible Points: _____________
Understandability Score (%): _____________
(Total Points / Total Possible Points x 100)

Actionability

	Item	Response Options	Rating
20	The material clearly identifies at least one action the user can take.	Disagree=0, Agree=1	
21	The material addresses the user directly when describing actions.	Disagree=0, Agree=1	
22	The material breaks down any action into manageable, explicit steps.	Disagree=0, Agree=1	
25	The material explains how to use the charts, graphs, tables, or diagrams to take actions.	Disagree=0, Agree=1, No charts, graphs, tables, diagrams=N/A	

Total Points: _____________
Total Possible Points: _____________
Actionability Score (%): _____________
(Total Points / Total Possible Points x 100)

Table S6. Standardized Data Extraction Form and Coding Rules

S6.1 Data extraction variables

**Variable**	**Operational definition**	**Format/coding**
Video URL	Original video link used for internal verification and traceability.	Text string (URL)
Video ID	Unique de-identified video code assigned by the researchers.	D001-D086; K001-K092; B001-B050
Platform	Source platform from which the video was retrieved.	See S6.2

Variable	Operational definition	Format/coding
Video URL	Original video link used for internal verification and traceability.	Text string (URL)
Video duration	Total length of the video.	Seconds
Days since upload	Number of days between the upload date and the data extraction date.	Numeric
Uploader followers	Number of followers displayed on the uploader profile at extraction.	Numeric
Likes	Number of likes displayed at extraction.	Numeric
Comments	Number of comments displayed at extraction.	Numeric
Saves	Number of times the video was saved or bookmarked by users; corresponding to the Chinese platform function "收藏".	Numeric
Shares	Number of shares displayed at extraction.	Numeric
Creator identity	Type of account responsible for posting the video.	See S6.2
Verification status	Platform-based account certification status.	See S6.2
Content theme	Dominant educational focus of the video.	See S6.2
Presentation format	Main form used to present the video content.	See S6.2
JAMA score	Transparency score based on the JAMA benchmark criteria.	0-4
GQS score	Overall educational quality score based on the Global Quality Scale.	1-5
mDISCERN score	Reliability score based on the modified DISCERN instrument.	0-5
PEMAT-understandability	Understandability score based on PEMAT-A/V.	0-100%
PEMAT-actionability	Actionability score based on PEMAT-A/V.	0-100%

S6.2 Coding rules for categorical variables

Variable	Code	Category	Operational definition and coding rule
Platform	1	Douyin	The short-form video platform Douyin (the Chinese version of TikTok).
Platform	2	Kwai	The Kuaishou platform, also known internationally as Kwai.
Platform	3	Bilibili	The Chinese video-sharing platform Bilibili.
Creator identity	1	Medical professionals	Individual accounts self-identifying as physicians, nurses, pharmacists, rehabilitation therapists, or other healthcare workers, with or without platform verification.
Creator identity	2	Healthcare organizations	Institutional accounts operated by hospitals, clinics, academic institutions, government health agencies, or professional medical organizations.
Creator identity	3	Independent users	Accounts without identifiable healthcare professional or institutional affiliation, including lay users, media creators, and non-clinical accounts.
Verification status	1	Individual verified (Yellow V)	Accounts with individual platform verification, typically assigned to certified professional or influential individual users.
Verification status	2	Organization verified (Blue V)	Accounts with institutional platform verification, typically assigned to hospitals, media, government agencies, or organizations.
Verification status	3	Unverified	Accounts without an official platform verification badge.

Variable	Code	Category	Operational definition and coding rule
Content theme	1	Disease knowledge	Videos mainly explaining COPD definitions, etiology, symptoms, risk factors, disease progression, prevention, or general disease-related knowledge. If personal experience was used primarily to explain general knowledge, the video was coded here.
Content theme	2	Clinical scenarios	Videos mainly depicting diagnosis, clinical consultation, acute exacerbation, hospitalization, case examples, doctor-patient interaction, or other clinical contexts.
Content theme	3	Intervention strategies	Videos mainly focusing on pharmacological treatment, inhaler use, pulmonary rehabilitation, breathing exercises, lifestyle modification, nursing care, or self-management behaviors.
Presentation format	1	Oral monologue	A speaker directly explains information to the audience, usually in a talking-head format, without a dominant dramatized scene or complex visual illustration.
Presentation format	2	Visual illustration	Information is mainly delivered through non-live-action visual elements such as animation, slides, diagrams, figures, or text-on-screen presentations.
Presentation format	3	Situational enactment	The video uses live-action contextual presentation, including dialogue, documentary-style clips, real-person demonstrations, simulated clinical scenarios, or skill demonstrations.

Note. Numeric codes were used for data entry and statistical analysis. Saves were treated as a platform-displayed engagement metric. Although the metric was harmonized across platforms, platform-specific interface designs may differ; therefore, saves should be interpreted as a comparable displayed engagement indicator. For videos containing multiple themes or presentation formats, coding was based on the dominant theme or format representing the primary educational purpose. When creator professional status could not be determined from profile information, platform verification, account description, or video content, the account was coded as an independent user. Fine-grained presentation subtypes were consolidated into three categories according to their dominant communication mode and shared visual or narrative characteristics.

S6.3 Quality assessment scoring record

The following blank scoring record was used for each included video to document rater-specific and final consensus scores for each assessment instrument. Final consensus scores were used for descriptive and inferential analyses, while rater-specific scores were retained internally for inter-rater and test-retest reliability analyses.

Assessment tool	Construct assessed	Score range	Rater 1	Rater 2	Rater 1 retest score	Final consensus score	Notes
JAMA benchmark criteria	Transparency and traceability of online health information.	0-4					Four benchmark items: authorship, attribution, disclosure, and currency.
GQS	Overall educational quality, flow, usefulness, and practical value.	1-5					Higher scores indicate better overall quality.

Assessment tool	Construct assessed	Score range	Rater 1	Rater 2	Rater 1 retest score	Final consensus score	Notes
mDISCERN	Reliability of health information.	0-5					Higher scores indicate greater reliability.
PEMAT-understandability	Ease with which patients can understand the audiovisual material.	0-100%					Higher percentages indicate greater understandability.
PEMAT-actionability	Extent to which the material supports patient action or behavior.	0-100%					Higher percentages indicate greater actionability.

S6.4 Standardized extraction form

Metadata and engagement fields

Video ID	Platform	Duration (s)	Days since upload	Followers	Likes	Comments	Saves	Shares

Coding and quality assessment fields

Creator identity	Verification status	Content theme	Presentation format	JAMA	GQS	mDISCERN	PEMAT-U	PEMAT-A
